# Aortic Balloon Valvuloplasty Prior to Orthotopic Liver Transplantation: A Novel Approach to Aortic Stenosis and End-Stage Liver Disease

**DOI:** 10.1155/2014/325136

**Published:** 2014-11-09

**Authors:** Edward Coverstone, Kevin Korenblat, Jeffrey S. Crippin, William C. Chapman, Andrew M. Kates, Alan Zajarias

**Affiliations:** ^1^Cardiovascular Division, Washington University School of Medicine, Campus Box 8086, 660 S. Euclid Avenue, St. Louis, MO 63110, USA; ^2^Gastroenterology Division, Washington University School of Medicine, Campus Box 8124, 660 South Euclid Avenue, St. Louis, MO 63110, USA; ^3^Transplant Division, Washington University School of Medicine, Campus Box 8109, 660 South Euclid Avenue, St. Louis, MO 63110, USA

## Abstract

The combination of severe aortic stenosis and end-stage liver disease increases the morbidity and mortality of surgical aortic valve replacement or orthotopic liver transplantation resulting in a prohibitive operative risk. We propose a staged approach of balloon aortic valvuloplasty prior to orthotopic liver transplantation as a bridge to definitive aortic valve replacement. Between 2010 and 2012, four patients with severe aortic stenosis and end-stage liver disease underwent staged balloon aortic valvuloplasty followed by orthotopic liver transplantation. All patients had been deemed to be inappropriate candidates for liver transplantation or aortic valve surgery due to their comorbidity. One patient died of complications from a perivalvular abscess. Three patients went on to successful graft implantation and function and surgical recovery. Two of the three patients proceeded to definitive surgical aortic valve replacement with the remainder currently undergoing evaluation. In this case series, we present a novel approach of balloon aortic valvuloplasty prior to liver transplantation as a potential bridge to definitive treatment of severe aortic stenosis in the end-stage liver patient.

## 1. Introduction

Severe aortic stenosis (AS) is associated with significant operative morbidity and mortality with risk of death or cardiovascular complication in noncardiac surgery patients estimated as high as 31% [1]. End-stage liver disease (ESLD) complicates perioperative hemodynamics causing peripheral vasodilatation, decreased cardiac afterload, and increased cardiac output generating poor surgical outcomes for cardiac surgery [2]. The risks of concomitant cardiovascular disease, such as severe AS, in orthotopic liver transplantation (OLT) have led the American Association for the Study of Liver Disease to recommend echocardiogram and stress testing for all transplant evaluations [3]. In our clinical experience, the combination of severe AS and ESLD makes transplantation unacceptably high surgical risk.

To address this risk, prior case reports describe both pretransplant and simultaneous cardiac surgery such as surgical aortic valve replacement (AVR) and orthotopic heart transplant [4]. In 2001, Parker et al. and Eckhoff et al. reported the first cases of combined aortic valve replacement and orthotopic liver transplantation with successful outcomes but raised concerns for prolonged surgical times leading excessive donor organ ischemia as well as issues with coagulopathy and fibrinolysis [5, 6]. Nishida et al. then described in 2003 a case of AVR and OLT complicated by intraoperative hemorrhage requiring excess transfusions, as well as postoperative shock from presumed pulmonary embolism [7]. Therefore, cardiac surgery prior to or concomitant with liver transplantation is not without significant risk for morbidity and mortality.

We propose that balloon aortic valvuloplasty (BAV) may play a role in severe AS patients preparing for liver transplantation. To our knowledge, we present the first descriptions in the literature of BAV in the AS and ESLD patient as a bridge to liver transplant and aortic valve replacement.

## 2. Case Presentation

From 2010 to 2012, four patients were referred to our Center for Valvular Heart Disease for preoperative evaluation for liver transplantation (Table 1). All underwent echocardiography that identified severe AS. One patient underwent BAV prior to referral to our institution. All underwent evaluation by cardiology and cardiothoracic surgery and were deemed to have unacceptably high risk for surgical aortic valve replacement.

Patient 1 was a 58-year-old female with primary biliary cirrhosis who presented with decompensated cirrhosis and hepatorenal syndrome necessitating inpatient transfer for semiurgent liver transplantation. Upon arrival, the patient was noted to have a murmur and transthoracic echocardiogram revealed a calcified, restricted aortic valve with aortic valve area (AVA) of 0.8 cm^2^.

Patient 2 presented after being denied liver transplantation listing at an outside academic institution. He was 63 years old with hereditary hemochromatosis as well as a bicuspid valve and symptomatic, severe AS. At a prior institution, the patient had undergone balloon valvuloplasty with improvement of symptoms and his aortic valve area to 1.2 cm^2^ as assessed by echocardiography. At our institution, he underwent repeat echocardiography and was listed for liver transplantation.

Patient 3 was a 56-year-old male with longstanding cryptogenic cirrhosis referred as an outpatient after liver transplantation evaluation identified exertional dyspnea and AS with an AVA of 1.0 cm^2^ by echocardiography.

Patient 4 was 58 years old with hepatitis C- and alcohol-induced liver disease resulting in multiple ICU stays for cirrhosis complications. He was noted to have a heavily calcified bicuspid aortic valve with AVA of 0.7 cm^2^ by echocardiography. His liver disease worsened with refractory ascites, encephalopathy, and hepatorenal syndrome requiring hemodialysis.

**Figure 1:**
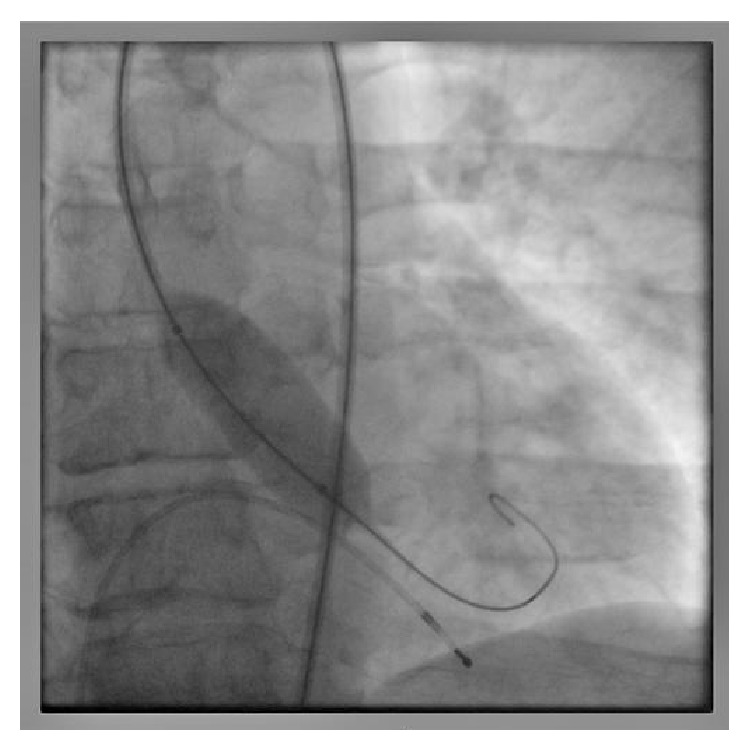
Balloon aortic valvuloplasty is performed under rapid ventricular pacing prior to liver transplantation.

**Figure 2:**
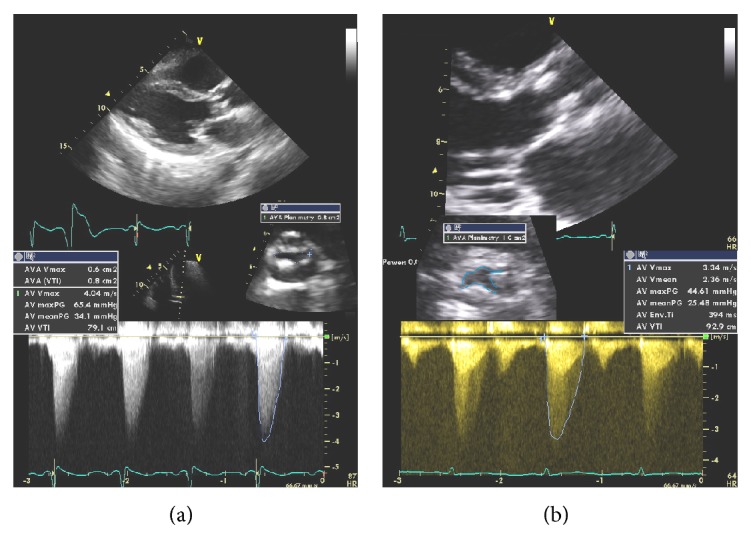
Transthoracic echocardiography before and after valvuloplasty shows a calcified, stenotic aortic valve with AVA of 0.8 cm^2^ by planimetry and continuity equation (VTI). Postvalvuloplasty there is demonstrated improvement in aortic valve gradients as well as AVA.

Patients 1, 3, and 4 were listed for transplant with plans for staged valvuloplasty less than 48 hours prior to OLT (Figure 1). Each underwent successful BAV with invasive and echo documentation of improvements in valvular gradients and AVA. (Figure 2) Each patient then underwent OLT within 38 hours of valvuloplasty.

All patients had successful graft function postoperatively. Patients 1, 2, and 3 weaned quickly from vasopressor and ventilator support and were discharged successfully from the hospital within fourteen days of transplant. Patient 4 underwent complicated operation marked by excessive blood loss and hypotension. Seven days postoperatively, he developed* S. epidermidis* bacteremia and an aortic root abscess requiring emergent open aortic valve replacement. He died six days later of pneumonia and respiratory failure.

Of the three surviving patients on follow-up, two went on to undergo successful open aortic valve replacement with the remaining patient planned for upcoming valve replacement surgery more than ten months after OLT.

## 3. Discussion

Definitive treatment of severe AS in patients with decompensated liver disease is marked by significant morbidity and mortality. Significant AS in the setting of fluid shifts, ventilator effects, and vasoactive medications during liver transplant places patients at high surgical risk [4]. Operative morbidity for cardiac surgery in the end-stage liver patient ranges from 50 to 100% [8]. Although data is lacking, transcatheter replacement via transapical or transaortic approach may carry similar morbidity to open surgery. Transcatheter aortic valve replacement via transfemoral approach may be an attractive future approach; however limited prior studies have shown increased mortality in patients with severe liver disease [9]. BAV prior to liver transplantation may be an option to reduce the cardiovascular risk of liver transplantation; however, the procedure is not without its own risks.

One patient in this series developed a serious complication of aortic root abscess. Multiple invasive procedures and his immunocompromised state were potential contributors to his susceptibility to bacteremia and infection. Although exceedingly rare, infective endocarditis has been described as a complication of BAV [10]. While the etiology was unclear in this case, bacterial infections remain the leading cause of mortality in end-stage liver disease and up to one-third of hospitalized cirrhotic patients have bacterial infections [11]. Given the increased susceptibility to infection, antimicrobial prophylaxis may be beneficial during the periprocedural time period [12].

Our center currently utilizes a planned approach to perform urgent, on-call valvuloplasty once a donor liver is identified. Should valvuloplasty be unsuccessful, a back-up recipient is always available. Echocardiographic and invasive measurements are utilized to verify optimal improvement in hemodynamics prior to proceeding to the operative room in order to guarantee utility of a scarce resource. Given the risk of major bleeding due to coagulopathy, sheath removal is performed surgically at the time of transplant or soon thereafter.

The management of the AS patient with concomitant liver disease also requires accurate diagnosis and severity assessment. The peripheral vasodilation and elevated cardiac output of ESLD result in high transvalvular flows velocities that may overestimate the degree of stenosis [13, 14]. Cirrhotic cardiomyopathy will mask left ventricular systolic dysfunction and induce diastolic dysfunction and left ventricular hypertrophy [15, 16]. Furthermore, left ventricular outflow tract obstruction has been described as a common finding among cirrhotic patients undergoing liver transplantation evaluation [17]. The careful use of dobutamine stress echocardiography, as well as transesophageal echocardiography and catheter-based assessments, may help to verify the severity of AS and distinguish entities such as pseudo AS and paradoxical low-flow, low-gradient AS [18–20]. The use of a gradient only based assessment of valve severity should be discouraged [14].

In conclusion, the current morbidity and mortality of surgical aortic valve replacement or orthotopic liver transplantation in patients with severe AS and ESLD may result in prohibitive operative risk. We present our developing approach as elucidated by four patients previously deemed to be inappropriate candidates for liver transplantation or aortic valve surgery due to their comorbidity. We conclude that in patients otherwise unsuitable for liver transplantation due to the severity of their AS, staged valvuloplasty and liver transplantation may be a practical approach that permits long-term, definitive valve replacement posttransplantation. Further investigation is needed to assess the safety and procedural success of this approach.

## Figures and Tables

**Table 1:** Patient and procedural characteristics before and after valvuloplasty.

	Patient 1		Patient 2		Patient 3		Patient 4	
Age, y/sex	58/female		63/male		56/male		58/male	
Diagnosis	Primary biliary Cirrhosis		Hemochromatosis		Nonalcoholic steatohepatitis		Hepatitis C	
Platelet count	101		101		24		38	
MELD score	38		28		16		36	
BNP	801		419		33		54	

Hemodynamic data	Pre	Post	Pre	Post	Pre	Post	Pre	Post

Gradients, mean (mmHg)	37	28	45	16	35	25	45	25
Aortic valve area (cm^2^)^†^	0.8	1.0	0.6	1.2	1.0	1.1	0.7	1.1
Ejection fraction	60%	64%	60%	70%	65%	70%	68%	65%

Procedure data								
Time to transplant	38 hrs		4 mo		6 hrs		20 hrs	
Balloon size	23 mm, CRISTAL		∗		23 mm, CRISTAL		23 mm, Z-MED II	
Sheath size	8 French		10 French		10 French		12 French	
Sheath removal	Surgical, time of transplant		—		Surgical, 48 hrs after transplant		Time of transplant	
Follow-up, mo/outcomes	24 mo, alive, s/p AVR		10 mo, alive		24 mo, alive, s/p AVR		Deceased, perivalvular abscess	

^†^Estimated by transthoracic echocardiographic measurement; continuity equation ^*^unrecorded.
